# Expansion of *BCR/ABL1* ^+^ cells requires PAK2 but not PAK1

**DOI:** 10.1111/bjh.14833

**Published:** 2017-07-14

**Authors:** Leo Edlinger, Angelika Berger‐Becvar, Ingeborg Menzl, Gregor Hoermann, Georg Greiner, Eva Grundschober, Zsuzsanna Bago‐Horvath, Wael Al‐Zoughbi, Gerald Hoefler, Christine Brostjan, Lars Gille, Richard Moriggl, Andreas Spittler, Veronika Sexl, Andrea Hoelbl‐Kovacic

**Affiliations:** ^1^ Institute of Pharmacology and Toxicology Department of Biomedical Sciences University of Veterinary Medicine Vienna Vienna Austria; ^2^ Department of Chemical and Physical Sciences University of Toronto Mississauga Mississauga ON Canada; ^3^ Department of Laboratory Medicine Medical University of Vienna Vienna Austria; ^4^ Clinical Institute of Pathology Medical University of Vienna Vienna Austria; ^5^ Institute of Pathology Medical University of Graz Graz Austria; ^6^ Department of Surgery Research Laboratories Medical University of Vienna Vienna General Hospital Vienna Austria; ^7^ Ludwig Boltzmann Institute for Cancer Research (LBI‐CR) Vienna Austria; ^8^ Institute of Animal Breeding and Genetics University of Veterinary Medicine Vienna Vienna Austria; ^9^ Core Facility Flow Cytometry & Department of Surgery Research Laboratories Medical University of Vienna Vienna Austria

**Keywords:** PAK2, *BCR/ABL1*, CML, lymphoma, p21‐activated kinases, exosomes

## Abstract

The p21‐activated kinases (PAKs) are key nodes in oncogenic signalling pathways controlling growth, survival, and motility of cancer cells. Their activity is increased in many human cancers and is associated with poor prognosis. To date, PAK deregulation has mainly been studied in solid tumours, where PAK1 and PAK4 are the main isoforms deregulated. We show that PAK1 and PAK2 are the critical isoforms in a *BCR/ABL1* ^+^ haematopoietic malignancy. In suspension, leukaemic cells deficient for PAK1 and PAK2 undergo apoptosis, while the loss of either protein is well tolerated. Transfer of medium conditioned by shPAK2‐ but not shPAK1‐expressing leukaemic cells interferes with endothelial cell growth. We found that leukaemic cells produce exosomes containing PAK2. Transfer of isolated exosomes supports endothelial cell proliferation. In parallel, we found that leukaemic cells explicitly require PAK2 to grow towards an extracellular matrix. PAK2‐deficient cells fail to form colonies in methylcellulose and to induce lymphomas *in vivo*. PAK2 might therefore be the critical isoform in leukaemic cells by controlling tumour growth in a dual manner: vascularization via exosome‐mediated transfer to endothelial cells and remodelling of the extracellular matrix. This finding suggests that the PAK2 isoform represents a promising target for the treatment of haematological diseases.

The p21‐activated kinases (PAKs) are conserved non‐receptor serine/threonine kinases integrating a variety of signalling pathways in biological processes, such as proliferation, apoptosis, cytoskeletal structure, and cellular motility (Hofmann *et al*, 2004; Kumar *et al*, 2006; Radu *et al*, 2014; Rane & Minden, 2014). PAKs are divided into group I PAKs (PAK1, PAK2, and PAK3) and group II PAKs (PAK4, PAK5, and PAK6) based on their sequence, function, and regulation. Originally, PAKs were discovered as effector proteins of the Rho GTPases Cdc42 and Rac1. However, in the last few decades, PAKs have gained increasing attention in tumour biology.

PAKs act downstream of oncoproteins as regulators of tumour‐specific signalling pathways (Hofmann *et al*, 2004; Kumar *et al*, 2006; Radu *et al*, 2014; Rane & Minden, 2014). In human cancer, PAK1 is the most frequently described and studied isoform (Kumar *et al*, 2006). Its increased activity can be caused by elevated protein levels, hyperactivation of GTPases, or downregulated expression of endogenous inhibitors (Kumar *et al*, 2006; Radu *et al*, 2014; Rane & Minden, 2014). Late stage tumours of tissues, such as brain, breast, ovary, pancreas, or colon, display increased PAK1 activity (Kumar *et al*, 2006). In a murine MCF‐7 breast cancer model, PAK1 promoted proliferation and anchorage‐independent growth (Adam *et al*, 2000). However, in recent years, mounting evidence suggests that other PAK members also contribute to tumour formation and progression (Kumar *et al*, 2006; Radu *et al*, 2014; Rane & Minden, 2014). Gene amplification or increased levels of PAK4 were found in pancreatic cancer cells and are linked to anchorage‐independent cancer‐cell growth (Mahlamäki *et al*, 2004).

Less is known about the role of PAK2 in cancer despite its pronounced ability to regulate apoptosis. In this regard, PAK2 is unique among PAK isoforms as it can positively or negatively influence apoptosis (Huang *et al*, 2003, 2004; Kumar *et al*, 2006; Radu *et al*, 2014). These latter properties qualify PAK2 as a tumour promoter.

PAK2 is a close homologue of PAK1. However, knockout mice revealed distinct functions for either kinase. Upon loss of PAK1, mice display reduced mast cell degranulation and disturbed glucose homeostasis (Allen *et al*, 2009; Wang *et al*, 2011; Rane & Minden, 2014). Mice deficient for PAK2 die on embryonic day E8.5 due to defects in endothelial development (Hofmann *et al*, 2004; Radu *et al*, 2015). In adult mice, conditional deletion of PAK2 led to increased vascular permeability (Radu *et al*, 2015).

In haematopoietic tumours, disease‐promoting roles have been implicated for PAK1 and PAK2. The level and the activity of PAK1 are increased in myelodysplastic syndrome (MDS) (Pandolfi *et al*, 2015), and a gain in *PAK1* gene copy number was found in cutaneous T cell lymphoma (mycosis fungoides/Sezary syndrome) (Mao *et al*, 2003). The level of PAK2 is increased in mantle cell lymphoma (MCL) and in activated B‐cell like diffuse large B cell lymphoma (ABC‐like DLBCL) that harbour 3q29 amplifications. The gain of 3q29 is associated with poor prognosis (Beà *et al*, 2004; Bea *et al*, 2005; Salaverria *et al*, 2007). In *BCR/ABL1* ^+^ leukaemia, PAK1 and PAK2 are upstream regulators of the transcription factor STAT5 – a key node for initiation and maintenance of disease (Hoelbl *et al*, 2006, 2010; Friedbichler *et al*, 2010; Berger *et al*, 2014).

Here, we studied the individual roles of PAK1 and PAK2 in *BCR/ABL1* ^+^ leukaemia. Using a combination of *in silico* and loss of function studies *in vitro* and *in vivo*, we identified PAK2 as the dominant PAK isoform controlling leukaemic cell survival.

## Materials and methods

### Mice

NSG mice (NOD.Cg‐*Prkdc* ^*scid*^ *Il2rg*tm^*1Wjl*^/SzJ; The Jackson Laboratory, Bar Harbor, ME, USA) were maintained at the University of Veterinary Medicine Vienna. All animal experiments were approved by the local ethics committee and conform to Austrian law (license BMWFW‐68.205/0112‐WF/V/3b/2016).

### Cell culture

The human *BCR/ABL1* ^+^ KU812 cell line was cultured in RPMI medium (Sigma‐Aldrich, St. Louis, MO, USA) supplemented with 10% fetal calf serum (FCS), 50 μmol/l 2‐mercaptoethanol, 100 u/ml penicillin, and 100 μg/ml streptomycin (PAA Laboratories GmbH, Pasching, Austria). Human Umbilical Vein Endothelial Cells (HUVEC) cells were cultured in M199 medium (Sigma‐Aldrich) supplemented with 20% FCS, 100 u/ml penicillin, 100 μg/ml streptomycin (PAA), 0·5 μg/ml amphotericin B, 2 u/ml heparin, 2 mmol/l l‐glutamine, and 100 μg/ml endothelial cell growth supplement. HEK293T packaging cells and murine embryonic fibroblasts (MEFs) were cultured in Dulbecco's modified Eagle's medium (DMEM; Sigma‐Aldrich) supplemented with 10% FCS, 100 u/ml penicillin, and 100 μg/ml streptomycin (PAA).

### shRNA‐mediated knockdown

Lentiviral shRNA vectors GIPZ (encoding shPAK1 and eGFP) and pLKO.1 (encoding shPAK2 and mCherry) were prepared according to the manufacturer's instructions (Thermo Scientific, Waltham, MA, USA). Two non‐targeting shRDM (RHS6848 and RHS4346) vectors were used as controls. The target sequences of the vectors are as follows: shPAK1_V3LHS_347245 (5′‐CCAGAGGTTGTGACACGAA‐3′), shPAK1_ V2LHS_152621 (5′‐CCAAGAAAGAGCTGATTAT‐3′), shPAK2_TRCN0000002115 (5′‐ CTCTAGGAACCAAAGTGATTT‐3′), and shPAK2_TRCN0000002116 (5′‐ CAGACCTCCAATATCACCAAA‐3′). HEK293T packaging cells were transfected with shRNA constructs as described before (Greiner *et al*, 2017). Virus‐containing supernatant was used to transduce *BCR/ABL1* ^+^ KU812 cells. Combined knockdown of PAK1 and PAK2 was obtained by serial transduction of KU812 cells.

### Subcutaneous transplantation of leukaemic cells

NSG mice were subcutaneously (s.c.) injected with 10^5^ KU812 cells (shRDM, shPAK1, shPAK2, shPAK1/2). After 3 weeks, tumour nodules were palpable and tumour size was measured every other day with a slide caliper and calculated using the formula: *a* × *b*/2 (*a*: length of tumour; *b*: tumour width). After 36 days, mice were sacrificed, and tumour weights were determined.

### Immunoblotting

Whole‐cell lysates were harvested as described previously (Berger *et al*, 2014). Proteins were separated on a 7% sodium dodecyl sulphate polyacrylamide gel and transferred to nitrocellulose membranes. The following antibodies were used for immunoblotting: HSC‐70 (B‐6) (SC‐7298), β‐Actin (AC‐15) (SC‐69879) (Santa Cruz Technology, Dallas, TX, USA), PAK1 (2602), PAK2 (2608), Alix (3A9) (2171), pCRKL^Y207^ (3181), pSTAT5^Y694^ (C11C5) (9359) (Cell Signaling, Danvers, MA, USA). Chemiluminescent visualisation of the bands was performed with a Chemidoc MP imaging system device after incubation of the membranes with Clarity Western enhanced chemiluminescence reagent (Bio‐Rad, Hercules, CA, USA).

### Imatinib treatment

KU812 cells (10^6^: 25 × 10^4^ cells/ml) were seeded in six‐well dishes. Imatinib tyrosine kinase inhibitor (Selleck Chemicals, Munich, Germany) was added at a 2‐μmol/l concentration (dissolved in 0·02% dimethyl sulfoxide, DMSO), while 0·02% DMSO served as negative control. Treated cells were incubated at 37°C and 5% CO_2_, harvested, washed with phosphate‐buffered saline (PBS), and immediately lysed as described previously (Schuster *et al*, 2007).

### Exosome isolation and flow cytometry

KU812 cells and MEFs were washed three times with PBS and then cultured for 36 h before exosome isolation. Conditioned media were harvested (120 ml) and centrifuged (480 × ***g*** for 5 min, 2000 × ***g*** for 10 min, 10 000 × ***g*** for 30 min, all steps at 4°C) as described previously (Théry *et al*, 2006; Ji *et al*, 2013). Crude exosomes were isolated by repeated ultracentrifugation (100 000 × ***g*** for 60 min at 4°C, twice) as described previously (Théry *et al*, 2006).

For flow cytometry, exosomes were labelled with anti‐CD63‐fluorescein isothicyanate (FITC) (561924; BD Biosciences, San Jose, CA, USA) and anti‐CD81‐allophycocyanin (APC) (561958; BD Biosciences) and analysed on a MoFlo Astrios device using Summit v6.2 software (Beckman Coulter, Brea, CA, USA). Before labelling, antibodies were centrifuged at 45 000 × ***g*** for 5 min to exclude antibody aggregates. For assessment of size calibration, silica beads (100, 500, and 1000 nm; Kisker Biotech, Steinfurt, Germany) with a refractory index near to biological material were recorded. 100 nm silica beads clearly discriminated from laser noise. For exosome analysis, events overlaying laser noise were excluded. An effect of swarm/coincidence was excluded as pooled, single‐stained exosomes did not show any signs of double positivity.

### Quantitative real‐time polymerase chain reaction and angiogenesis array

RNA was isolated from KU812 (shRDM, shPAK1, shPAK2) cells using the peqGOLD TriFast reagent (Peqlab, Erlangen, Germany). RNA was transcribed with the iSCRIPT cDNA synthesis kit (Bio‐Rad). Quantitative real‐time PCR was performed on a MyiQ2 cycler (Bio‐Rad) with SsoAdvanced SYBR GreenSupermix (Bio‐Rad). Following primers were used: human *PAK1*: fwd (5′‐3′) GCTGTTCTGGATGTGTTGGA, rev (5′‐3′) TTCTGAAACTGGTGGCACTG, human *PAK2*: fwd (5′‐3′) TGAGCAGAGCAAACGCAGTA, rev (5′‐3′) GTACAAGGCCCTCAAGGGAT, and human *RPLP0*: fwd (5′‐3′) GAGGGTGTCCGCAATGTT, rev (5′‐3′) TTGACCTTTTCAGCAAGTGGGAAG. Target gene expression was normalized to *RPLP0*.

For the evaluation of deregulated angiogenic factors, we applied a RT² Profiler™ PCR Array Human Angiogenic Growth Factors (PAHS‐072Z; Qiagen, Hilden, Germany) according to the manufacturer's instructions.

### Growth curve

High‐purity sorted cells were plated on a 6‐well dish. Cell numbers were counted using a haemocytometer.

### Histology

Subcutaneous tumours were paraformaldehyde‐fixed, paraffin‐embedded, and stained with Haematoxylin/Eosin and anti‐Cleaved Caspase‐3 (D175) (9661; Cell Signaling). The sections were scanned and photographed with a Zeiss AxioImager Z1 (Zeiss, Oberkochen, Germany). Quantification was performed using HistoQuest software (TissueGnostics GmbH, Vienna, Austria) and GraphPad Prism software (GraphPad Software, Inc., San Diego, CA, USA). For the labeling of CD31, sections were incubated with an anti‐CD31 antibody (MA1‐40074; Thermo Scientific). Visualization was performed using an Envision horseradish peroxidase (HRP) Polymer System (Dako, Santa Clara, CA, USA). Analyses were performed with a Nikon Eclipse 80i microscope (Nikon, Amsterdam, The Netherlands) supplied with camera. Images were captured using NIS‐Elements D software (Nikon). For blood vessel density evaluation, labelled sections were scanned using ScanScope^®^ AT System (Aperio, Vista, CA, USA) and CD31 positive tumour blood vessels area was calculated using Definiens Tissue Studio software (Definiens, Munich, Germany).

### Scratch assay

HUVEC cells (5 × 10^5^) were seeded in 6‐well dishes coated with 2% gelatine. In parallel, 10^6^/ml KU812 cells were seeded without FCS and grown overnight. On the next day, supernatants of KU812 cells were collected and poured on HUVEC cells. A plastic pipette tip was used to scratch the confluent monolayer. Gap closure was monitored every 12 h using a CKX41SF microscope and a DP21 digital camera (both from Olympus, Hamburg, Germany). Quantification of the invaded area was performed with ImageJ software (NIH, Bethesda, MD, USA). For the scratch assay in the presence of isolated exosomes, exosomes derived from 2 × 10^8^ KU812 cells were administered to 10^5^ HUVEC cells in 12‐well dishes. Exosome‐free supernatant was harvested after the first ultracentrifugation.

### Soft‐agar assay

10^3^ KU812 cells were seeded in methylcellulose without supplement of cytokines (MethoCult, 03231; STEMCELL Technologies, Vancouver, BC, Canada). Colonies were counted after 14 days and photographed using a CKX41SF microscope and a DP21 digital camera (both from Olympus). Single colonies were picked for quantitative real‐time polymerase chain reaction (qPCR).

### Flow cytometry analysis, antibodies, and cell sorting

Single‐cell suspensions were analysed by a BD FACS Canto II flow cytometer equipped with 488, 633, and 405 nm lasers using FACS Diva software (Becton Dickinson, Franklin Lakes, NJ, USA) as described before (Berger *et al*, 2014). Propidium iodide (PI) and apoptosis stainings were performed as described previously (Berger *et al*, 2014) and according to manufacturer's instructions (Annexin V Apoptosis Detection Kit eFluor^®^ 450, 88‐8006; eBioscience, San Diego, CA, USA). eGFP^+^ or mCherry^+^ KU812 cells were high‐purity FACS sorted on a FACS Aria III equipped with a 488 nm laser at 4°C (Becton Dickinson). Exosomes were stained with anti‐CD63‐FITC (561924; BD Biosciences) and anti‐CD81‐APC (561958; BD Biosciences).

### Bioinformatic analysis

Bioinformatic analyses were performed with the publicly available database SurvExpress (http://bioinformatica.mty.itesm.mx:8080/Biomatec/SurvivaX.jsp; Aguirre‐Gamboa *et al*, 2013). Patients suffering from different haematological disease entities were divided into low‐ and high‐risk groups according to Aguirre‐Gamboa *et al* (2013), and levels of *PAK1* and *PAK2* expression were assessed. Expression of *PAK1‐6* was assessed with the publicly available software Genevestigator (https://genevestigator.com/gv/; Hruz *et al*, 2008). Only disease categories with ≥20 samples were included, and the top 20 hits were shown.

### Statistical analyses

Student's *t*‐test, one‐way anova (followed by Tukey multiple comparison test), and assessment of half maximal effective concentration (EC50) values were performed using GraphPad Prism software version 5.04 and 6.02 (GraphPad Software, Inc.). Statistical significance is indicated for each experiment specifically (*P < 0·05; **P < 0·01; ***P < 0·001).

## Results

### Elevated levels of PAK1 and PAK2 in high‐risk groups of haematological patients

**Figure 1:**
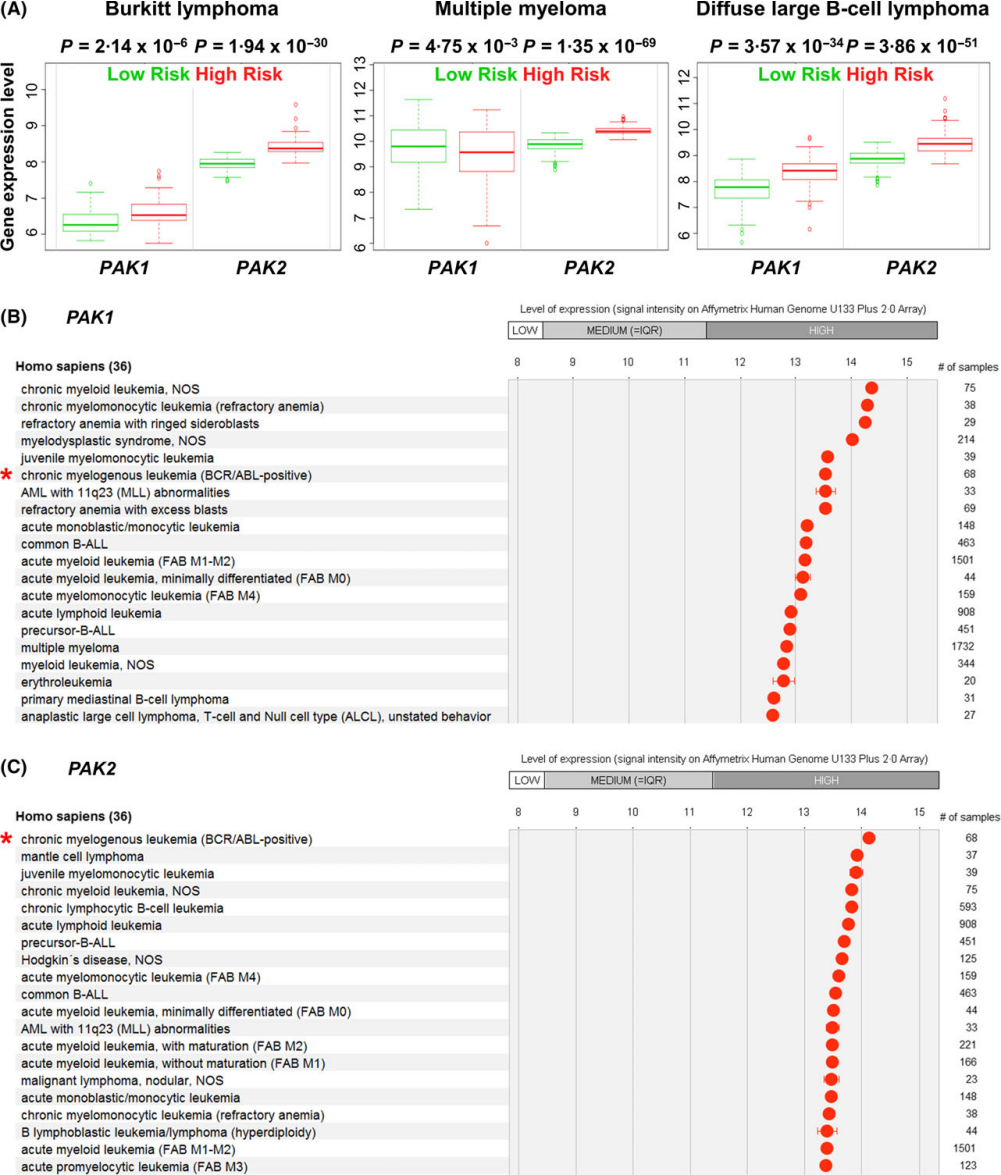
Levels of *PAK1* and *PAK2* in low‐ and high‐risk groups. (A) Expression of *PAK1* and *PAK2* in low‐ and high‐risk patients suffering from Burkitt lymphoma, multiple myeloma, and diffuse large B‐cell lymphoma [SurvExpress database (Aguirre‐Gamboa *et al*, 2013)]. *P*‐values indicate statistical significance in expression between low‐ and high‐risk groups. (B, C) Expression of *PAK1* and *PAK2* in haematological diseases according to the Genevestigator database (Hruz *et al*, 2008). Asterisks mark samples of *BCR/ABL1* ^+^ chronic myeloid leukaemia. BCR/ABL:*BCR/ABL1* fusion gene; MLL: mixed‐lineage leukaemia gene, now termed *KMT2A;* NOS: not otherwise specified.

We performed *in silico* analyses of a publicly available database providing expression data of patients that are assigned to a low‐ or a high‐risk group (SurvExpress; http://bioinformatica.mty.itesm.mx:8080/Biomatec/SurvivaX.jsp; Aguirre‐Gamboa *et al*, 2013). SurvExpress data revealed that levels of *PAK2* are significantly elevated in high‐risk groups of patients suffering from Burkitt lymphoma (BL), multiple myeloma (MM), diffuse large B‐cell lymphoma (DLBCL), and mantle cell lymphoma (MCL) (Figs 1A and S1A). The significances of expressions between low‐ and high‐risk groups are more pronounced with regard to levels of *PAK2* than to *PAK1* (the *P*‐values differing 1·10 × 10^24^ fold (BL), 3·25 × 10^66^ fold (MM), and 9·25 × 10^16^ fold (DLBCL); Fig 1A). In contrast, and in line with the proposed role of PAK1 in AML pathogenesis, levels of *PAK1* are significantly upregulated in a high‐risk group of AML patients (Pandolfi *et al*, 2015), whereas levels of *PAK2* are downregulated (Fig S1B).

As expression data in chronic myeloid leukaemia (CML) or acute lymphoblastic leukaemia (ALL) are not covered by the SurvExpress database, we switched to a different platform (Genevestigator; https://genevestigator.com/gv/; Hruz *et al*, 2008) which provides data of *BCR/ABL1*‐driven diseases. This platform allows the ranking of haematopoietic diseases based on the expression level of a given gene. Diseases with a sample size of at least 20 were included in the analysis. Some observations from the SurvExpress database were recapitulated: MM and MCL are identified among the top 20 diseases ranked according to levels of *PAK1* or *PAK2*. Of note, *PAK1* and *PAK2* [but not *PAK3*,* PAK4*,* PAK5* (*PAK7*), and *PAK6*] are highly expressed in CML and B‐ALL (Figs 1B,C and S2A–D). These *in silico* analyses indicate a privileged role of PAK1 and/or PAK2 in the pathogenesis of *BCR/ABL1*‐driven diseases and prompted us to investigate the consequences of PAK1 and PAK2 loss in a *BCR/ABL1* ^+^ disease model.

### Combined loss of PAK1 and PAK2 blocks leukaemic cell growth *in vitro*

**Figure 2:**
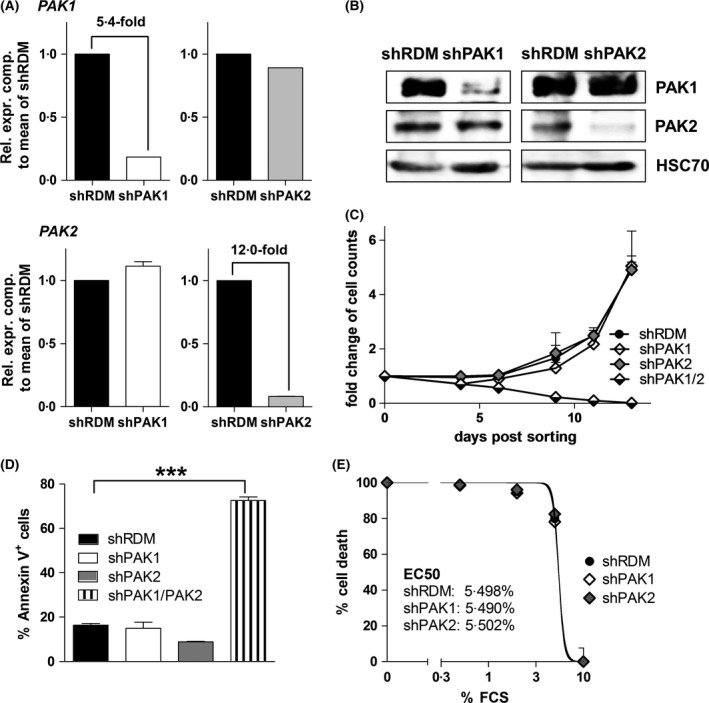
Combined knockdown of *PAK1* and *PAK2* leads to cell death in human *BCR/ABL1* ^+^ KU812 cells. (A) Knockdown of *PAK1* or *PAK2* in KU812 cells confirmed by qPCR (*n* = 3). Rel. expr. comp.: Relative expression compared. (B) Immunoblotting of KU812 cells expressing shRDM, shPAK1, or shPAK2. HSC70 served as loading control. (C) Growth curves of KU812 cells expressing shRDM, shPAK1, shPAK2, or shPAK1/2 (experiment in triplicates). (D) Frequencies of Annexin V^+^ cells determined by FACS on day 11 after high‐purity sorting of vector‐positive cells (*n* = 3). Graphs represent means ± SEM. (E) Serum withdrawal of KU812 cells expressing shRDM, shPAK1, or shPAK2 (*n* = 3). FCS, fetal calf serum.

We used the *BCR/ABL1* ^+^ KU812 cell line, a widely used system for the study of molecular mechanisms underlying CML pathogenesis (Kishi, 1985; Blom *et al*, 1992). We deliberately embarked on a CML and not an ALL cell line model as *PAK1* and *PAK2* expression was more prominently upregulated in CML (Fig 1B and C). To test whether *BCR/ABL1* interferes with PAK1 or PAK2 expression, we treated KU812 cells with the BCR/ABL1 kinase inhibitor Imatinib. No changes in PAK1 and PAK2 levels were detectable (Fig S3). Stable knockdown of *PAK1*,* PAK2*, or both genes was achieved using an established, miR30‐shRNA‐based system (Zuber *et al*, 2011; Fellmann *et al*, 2013; Putz *et al*, 2013, 2014; Berger *et al*, 2014; Scheicher *et al*, 2015). Knockdown efficiencies were verified by qPCR and immunoblotting (Fig 2A and B). For double knockdown, KU812 cells were first transduced with an shPAK1 construct, then high‐purity sorted, and finally transduced with an shPAK2‐encoding construct. Whereas KU812 cells either expressing shPAK1 or shPAK2 readily grew out, we failed – despite repeated efforts – to generate KU812 cells that tolerated knockdown of both *PAK1* and *PAK2* (Fig 2C). Apoptotic cells were significantly increased upon *PAK1/2* double knockdown (Fig 2D).

Cell cycle progression of shPAK1 and shPAK2 KU812 cells was assessed by propidium iodide (PI) staining and revealed a significantly reduced S‐phase in shPAK2 cells (Fig S4A and B). This defect did not translate into a different kinetic when performing growth curves (Fig 2C). To test whether the loss of PAK1 or PAK2 sensitizes cells for apoptosis, we performed a serum withdrawal assay. shPAK1‐, shPAK2‐, or random shRNA‐(shRDM‐)expressing KU812 cells were subjected to decreasing doses of FCS, and the frequency of cell death was determined. No changes were observed upon loss of PAK1 or PAK2 (EC50 of shRDM: 5·498%, shPAK1: 5·490%, shPAK2: 5·502%) (Fig 2E). To sum up, our observations indicate that the individual loss of PAK1 or PAK2 is well tolerated whereas the combined loss of both proteins is incompatible with survival of *BCR/ABL1* ^+^ cells *in vitro*.

### PAK2 but not PAK1 is required for growth in a factor‐free soft agar

**Figure 3:**
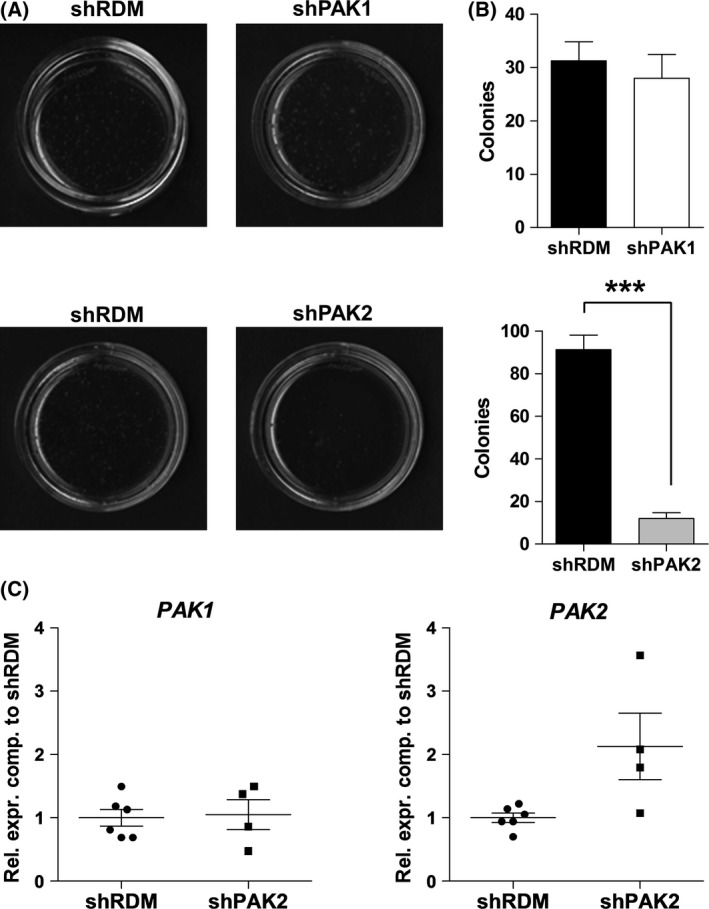
*PAK2* knockdown decreases colony formation. (A) Colony formation assays of KU812 cells expressing shRDM, shPAK1, or shPAK2 (*n* = 4). (B) Quantification of colonies depicted in (A). Graphs represent means ± SEM. (C) Expression of *PAK1* and *PAK2* in single shPAK2^+^ colonies. Rel. expr. comp.: Relative expression compared.

PAKs have been well described to integrate signals from the environment to mediate cell adhesion, cytoskeletal architecture, and proliferation (Hofmann *et al*, 2004; Kumar *et al*, 2006; Li *et al*, 2011; Radu *et al*, 2014; Rane & Minden, 2014). We tested the requirement for PAK1 and PAK2 in colony formation and performed methylcellulose assays. To obviate a potential mechanism that might counter‐regulate efficient knockdown, we performed these experiments immediately after sorting of shPAK1‐ or shPAK2‐expressing cells. Colony numbers of shPAK2‐expressing cells were significantly reduced after 14 days (Fig 3A and B). In contrast, the knockdown of *PAK1* resulted in a mild decrease of colony numbers, which failed to reach the level of significance (Fig 3B). We noted that the few colonies in the shPAK2 setup had regained *PAK2* expression (Fig 3C). These data suggest that PAK2 is required for growth in a soft‐agar assay, an effect that cannot be compensated for by PAK1. Only upregulation of PAK2 in an shPAK2 background allows colony formation.

### Loss of PAKs in leukaemic cells affects endothelial cell growth/proliferation

**Figure 4:**
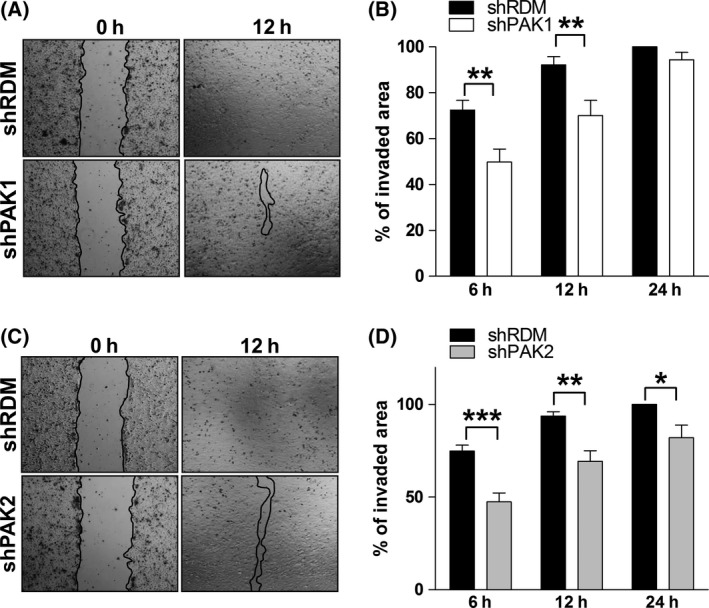
Knockdown of *PAK2* in human *BCR/ABL1* ^+^ KU812 cells interferes with endothelial cell growth. (A, C) Representative images of wound‐healing assays. Widths of scratches are indicated by black lines. (B, D) Quantification of the invaded area at indicated time points. Graphs represent means ± SEM (*n* = 6).

PAKs have been implicated in angiogenesis (Radu *et al*, 2014, 2015), and we tested whether PAK1 or PAK2 in leukaemic cells influences surrounding endothelial tissue. We performed an *in vitro* wound‐healing assay using human endothelial cells (HUVEC). HUVEC cells were grown to confluency, harmed by a scratch, and allowed to recover in the presence of conditioned medium (derived from KU812 cells expressing either the shPAK1 or the shPAK2 construct). The presence of ‘shPAK1 supernatant’ interfered with healing of the scratch after 6 and 12 h, but not after 24 h (Fig 4A and B). The effect of the ‘ shPAK2 supernatant’ was even more pronounced as no efficient scratch healing was achieved within 24 h (Fig 4C and D). From these data, we conclude that PAK2 and, to a lesser extent, PAK1 expression in leukaemic cells controls/delays the proliferation of surrounding endothelial cells, suggesting an *in vivo* role in tumour angiogenesis.

### KU812 cells produce exosomes that contain PAK2

To understand how leukaemic cell‐derived PAK expression impacts on endothelial cell growth via their supernatant, we tested the following ideas: First, PAK2 might regulate the transcription of angiogenic factors, which are secreted into the supernatant and interfere with endothelial cell proliferation. Alternatively, PAK2 may be contained in exosomes derived from KU812 cells, which fuse with endothelial cells. Thereby, PAK2 might directly regulate angiogenesis as described in a different cellular system (Gopal *et al*, 2016).

To test the first hypothesis, we performed a gene expression array that covered 84 genes involved in angiogenesis. We found a significant number of genes that interfere with angiogenesis or vascularization to be upregulated in shPAK2‐expressing KU812 cells (such as *TIMP1*,* BTG1*,* THBS1*,* IL12A*,* IL12B,* or *TIMP4*; Fig S5). We also found downregulation of e.g. the angiogenic sprouting promoting factor *TNF* (data not shown).

**Figure 5:**
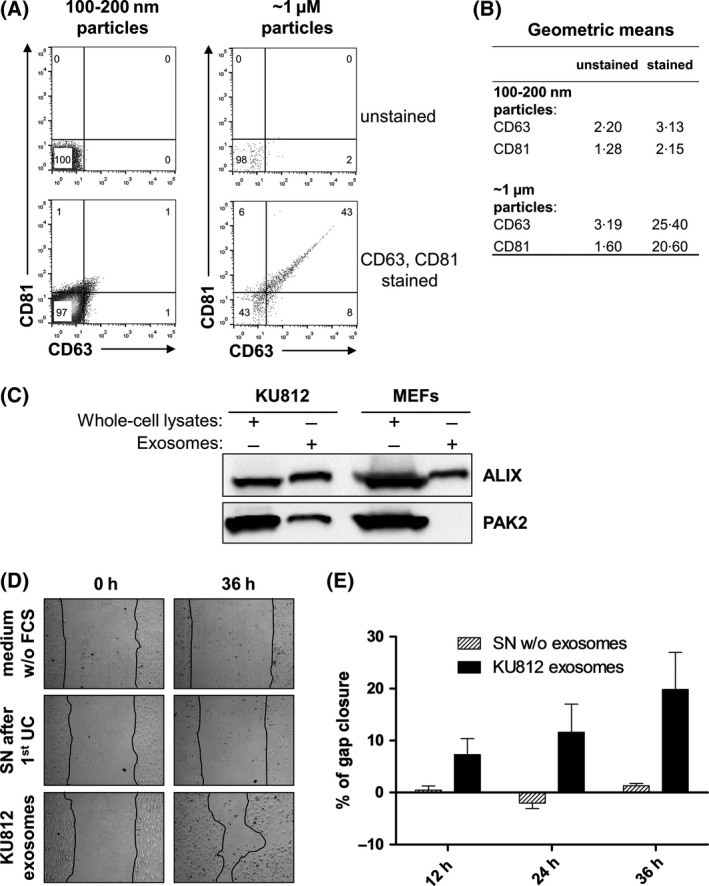
Human *BCR/ABL1* ^+^ KU812 cells produce exosomes that carry PAK2. (A) Flow cytometry of isolated exosomes (ultracentrifugation‐based method). Single exosomes (100–200 nm particles) and clusters of exosomes (~1 μm particles) were stained for exosome markers CD63 and CD81. (B) Geometric means of unstained and CD61 or CD83 positive populations in both fractions. (C) Immunoblotting of isolated exosomes for the exosome marker ALIX and for PAK2. (D) Representative pictures of wound‐healing assays in the presence of isolated exosomes (ultracentrifugation‐based method) or exosome‐deprived supernatant. Widths of scratches are indicated by black lines. (E) Quantification of the invaded area at indicated time points. Graphs represent means ± SEM (*n* = 3). FCS, fetal calf serum; SN, supernatant; UC, ultracentrifugation; w/o, without.

In parallel, we investigated whether KU812 cells produce exosomes. Exosomes were isolated using a standard ultracentrifugation‐based method, and their presence was verified using two different approaches. We pioneered and detected KU812 cell‐derived exosomes using a high resolution flow cytometer that allows resolving particles down to 0·1 μm. Exosomes either occur in isolation (size of 30–150 nm) or as clusters (Zomer *et al*, 2010; Paggetti *et al*, 2015). In line with this concept, we assessed exosome marker expression (CD81, CD63) on two different populations: particles of 100–200 nm and of ~1 μm size (Fig 5A). We identified positive staining in both populations. As the shifts of the ‘100–200 nm particle’ fraction were mild (most presumably a result of lower possible fluorescence intensity per particle due to the small size), we also assessed geometric mean values (Fig 5B). Geometric means of CD63 and CD81 expression shifted significantly compared to unstained exosomes. A complete set of controls is available in Fig S6.

The identity of the isolated exosomes was also confirmed by immunoblotting for the exosome marker ALIX. Of note, we detected that these exosomes contain PAK2 (Fig 5C). In line with the concept that PAK2 confers proliferation of endothelial cells, we found that these PAK2‐containing exosomes accelerate scratch healing, whereas exosome‐deprived supernatant hardly showed any effect (Fig 5D and E).

### PAK2 but not PAK1 is required for lymphoma growth in mice

**Figure 6:**
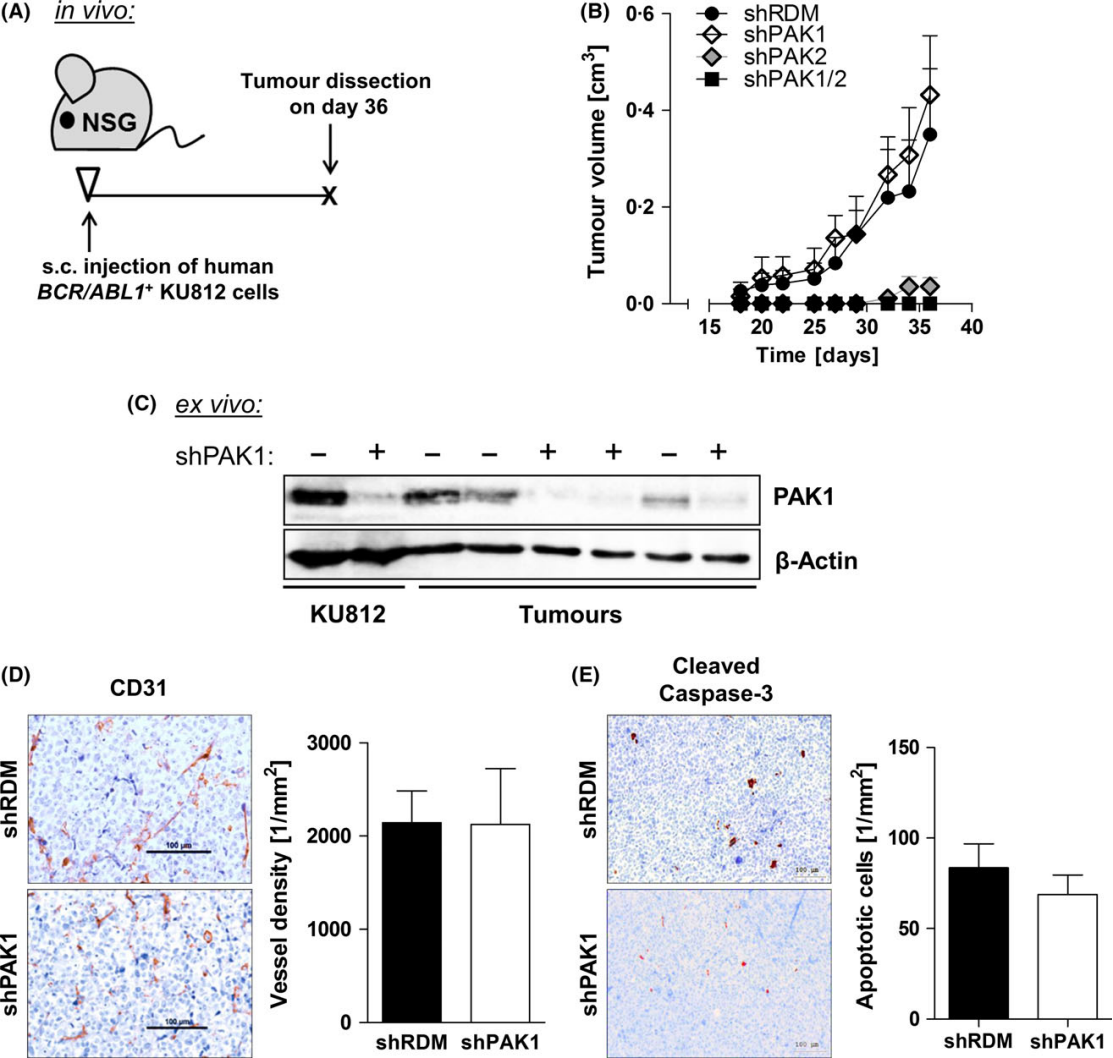
Loss of *PAK2* abrogates tumour formation of human *BCR/ABL1* ^+^ KU812 cells *in vivo*. (A) Scheme of experimental setup. Mice were injected subcutaneously with 10^5^ KU812 cells per flank, and solid tumours were dissected after 36 days. (B) Tumour volumes over a period of 36 days. (C) Protein levels of PAK1 in *ex‐vivo* derived tumour samples as determined by immunoblotting. (D) Immunohistochemical staining for CD31 (blood vessel density) in subcutaneous tumours. (E) Immunohistochemical staining for Cleaved Caspase‐3 in subcutaneous tumours.

We performed *in vivo* studies and used NOD.Cg‐*Prkdc* ^*scid*^ *Il2rg* ^*tm1Wjl*^/SzJ (NSG) mice in a xenotransplantation setting (Fig 6A). NSG mice lack the adaptive immune system, permit engraftment of human cells, and are ideally suited for studying tumour‐cell intrinsic effects *in vivo* (Shultz *et al*, 2005, 2007). High‐purity sorted shRDM‐, shPAK1‐, shPAK2‐, or shPAK1/2‐expressing KU812 cells were injected subcutaneously. Tumour volume was measured every 2–3 days over a period of 36 days. Tumour size in mice that had received shRDM‐ or shPAK1‐expressing cells gradually increased up to 0·35 ± 0·14 cm³ (shRDM) / 0·43 ± 0·12 cm^3^ (shPAK1) within 36 days. In line with the inability to form stable cell lines *in vitro*, the shPAK1‐expressing cells lost their tumourigenic potential upon additional loss of PAK2 (Fig 6B). The single loss of PAK2 sufficed to interfere with tumour development (Fig 6B). However, after a latency of 32 days, small tumours (0·01 ± 0·01 cm³) arose (0·04 ± 0·02 cm³ after 36 days) in the shPAK2 cohort. shPAK1/2‐expressing KU812 cells failed to form tumours. Hence, we failed to harvest biological material to perform further studies on histology or expression levels. We proceeded with the shRDM and shPAK1 cohorts for further analyses. Levels of PAK1 remained low throughout tumour development *in vivo* (Fig 6C). Histological sections of tumours showed comparable levels of blood vessel density in the shRDM cohort (2144 ± 341/mm²) and the shPAK1 cohort (2126 ± 598/mm²) as determined by CD31 staining (Fig 6D). In addition, there was no significant difference with regard to apoptotic cells within tumour tissue between shRDM (84 ± 13/mm²) and shPAK1 cohorts (69 ± 11/mm²) (Fig 6E).

## Discussion

Our study identifies PAK2 as key molecule for tumour cell growth and proliferation of endothelial tissue. Expression of PAK2 in leukaemic cells facilitates tumour development *in vivo*, formation of colonies *in vitro*, and endothelial cell proliferation. These abilities are not shared by its homologue PAK1.

Two independent sets of data support the unique role of PAK2 in tumourigenesis. PAK2‐deficient KU812 cells failed to grow in growth factor‐free soft agar – the few arising colonies showing compensatory upregulation of *PAK2*. Secondly, lymphoma formation *in vivo* was significantly impaired upon loss of PAK2. The defect in colony formation could be explained in two ways. KU812 cells require a certain cytokine or growth factor concentration that is continuously provided in suspension culture but not in a semi‐solid soft agar system. Alternatively, they are independent from extracellular factors but express factors that ‘cut’ the extracellular matrix (ECM) to allow expansion and colony formation. The latter concept is in line with the described roles of PAK1 and PAK2 in ECM remodelling: PAK1 enhances the expression of matrix metalloproteinase (MMP)1 and MMP3 in breast cancer cells and thereby supports the degradation of the extratumoural matrix (Rider *et al*, 2013). PAK2 increases the motility of ovarian cancer cells by enhancing breakdown of collagen type I (Flate & Stalvey, 2014). Our data support the latter concept because inhibitors for metalloproteinases (*TIMP1*,* TIMP4*) are increased upon knockdown of *PAK2*.

Our observation regarding the pivotal role of PAK2 in colony formation is shared by other groups studying different tumour types. Human prostatic adenocarcinoma cells and skin epidermal cells that are subjected to epidermal growth factor‐induced transformation show reduced colony formation upon loss of PAK2 (Li *et al*, 2011; Jiang *et al*, 2015).

Tumour‐derived PAK2 may act in a dual manner: it provides angiogenic factors by intracellular signalling, and it is shipped from the tumour to the endothelium via exosomes. Our study might unite two previous and independent observations: (i) leukaemic cells form exosomes (as shown for B‐CLL cells, Paggetti *et al*, 2015) and (ii) PAK2 can be packaged into exosomes (as shown for MDCK cells, Gopal *et al*, 2016). We show here, for the first time, that the CML cell line KU812 also produces exosomes and that PAK2 is packaged into these particles. We show that the administration of these particles then mobilizes endothelial cells and increases gap closure.

Of note, the defect in colony formation and in mobilization of endothelial cells translated to the inability to form solid tumours *in vivo*. This parallelism suggests that results derived from a soft‐agar assay are predictive for effects on tumour formation *in vivo* and underscores the importance of soft‐agar assays as screening models for lymphomagenesis. In methylcellulose, important features of tumour cells are requested: the breakdown of the ECM and invasion into the surrounding tissue, which is not necessary when cells grow in suspension culture.

Our data suggest that PAK2 (but not PAK1) is required for these processes in haematopoietic tumour cells. The significance of PAK2 might be explained by its involvement in multiple pathways with the potential to drive ECM remodelling and motility of cancer cells, including those featuring cytoskeletal effector proteins (Misra *et al*, 2005; Kumar *et al*, 2006; Chen *et al*, 2009; Flate & Stalvey, 2014; Radu *et al*, 2014).

Without the challenge of a surrounding matrix, PAK1 and PAK2 appear to compensate for each other. Cell cycle progression is mildly affected upon loss of PAK2, which is in line with a reduced mitotic timing (Jiang *et al*, 2015). However, this defect did not translate into differences in growth curve kinetics, suggesting compensation by PAK1. Consistent with this interpretation, the combined loss of PAK1 and PAK2 abrogated leukaemic cell growth *in vitro*. The unique role of PAK2 in haematopoietic tumour development only becomes apparent upon growing towards the resistance of a surrounding tissue.

The presence of PAK2 regulates haematopoietic tumour growth *in vivo* and cannot be compensated for by PAK1. This observation indicates that a compensatory upregulation of PAK1 upon a PAK2‐directed therapy will not interfere with therapeutic success. It may be valuable to assess the mutational status and levels of *PAK2* in patients suffering from haematopoietic diseases, focussing on lymphomas. Over‐activation of PAK2 might be indicative of an invasive, highly aggressive phenotype. A better understanding of the individual roles of PAK proteins in haematopoietic tumours may foster the development of precision medicine strategies.

## Author contributions

L.E. and A.BB. contributed equally to the study. L.E., A.BB., L.G., V.S., A.HK. designed the study. L.E. and A.BB. performed the experiments and collected the data. L.E., A.BB., I.M., Z.BH., W.AZ., A.S., V.S., A.HK. analysed and interpreted the data. G. Hoermann, G.G., E.G., G. Hoefler, C.B., L.G., R.M., A.S. provided essential resources. R.M. and A.S. critically revised the paper. L.E., A.BB., V.S., A.HK. wrote the manuscript and critically revised the paper.

## Competing interests

The authors have no competing interests.

## Supporting information


**Fig S1.** Expression of *PAK1* and *PAK2* in MCL and AML.

**Fig S2.** Expression of *PAK3*,* PAK4*,* PAK5 (7)*, and *PAK6* in haematological diseases.

**Fig S3.** Imatinib treatment of human *BCR/ABL1* ^+^ KU812 cells.

**Fig S4.** Cell cycle analysis of human *BCR/ABL1* ^+^ KU812 cells.

**Fig S5.** Array for genes regulating angiogenesis.

**Fig S6.** Controls for setup of flow cytometry (exosome detection).
