# Supplementary figures and images for: Expansion of BCR/ABL1 + cells requires PAK2 but not PAK1
Source: Br J Haematol. 2017 Jul 14;179(2):229–41. doi: 10.1111/bjh.14833 (PMC5655792; doi:10.1111/bjh.14833)

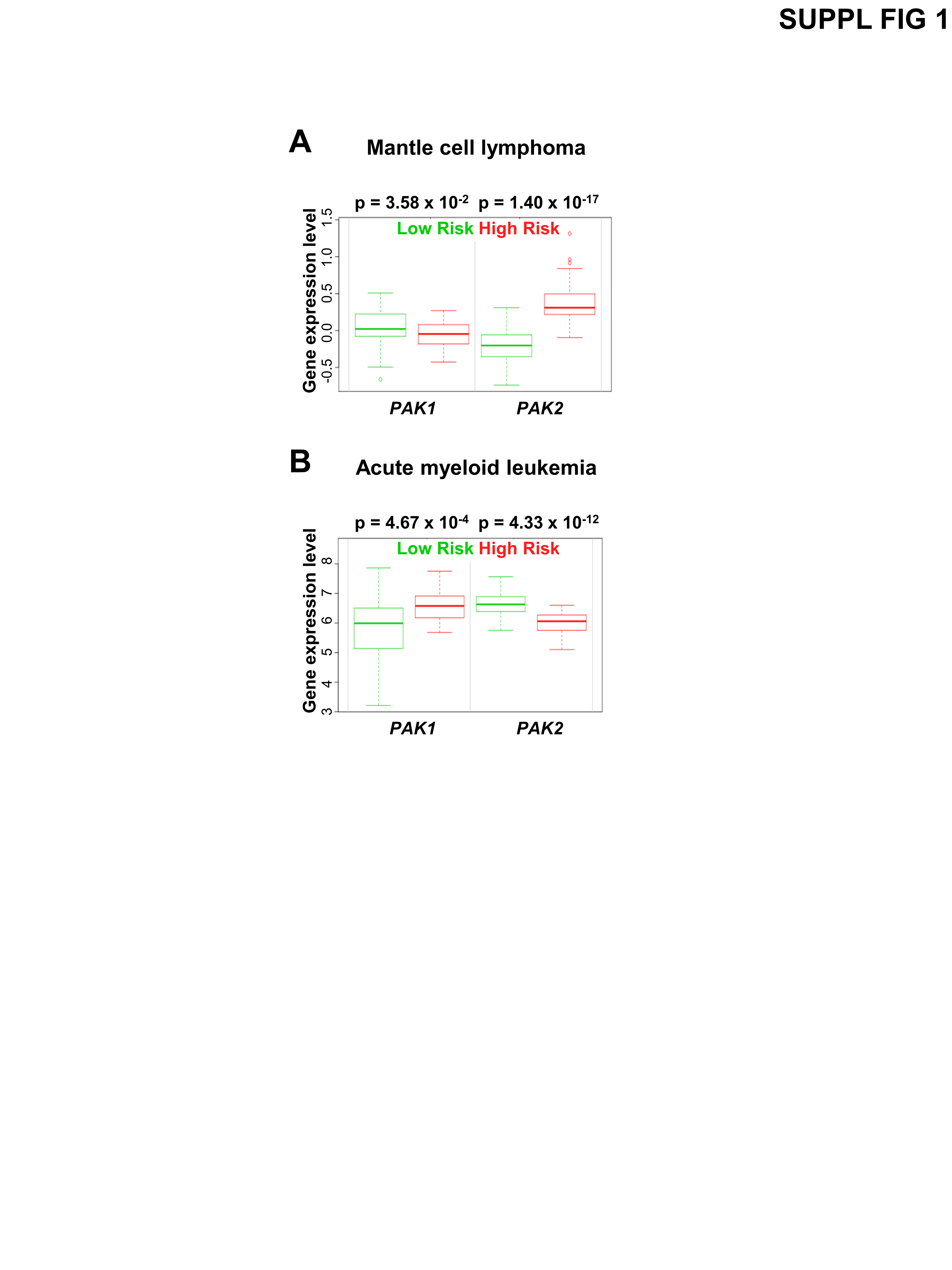

Supplement: Supplementary file 1 — Fig S1. Expression of PAK1 and PAK2 in MCL and AML. [file BJH-179-229-s001.tif]

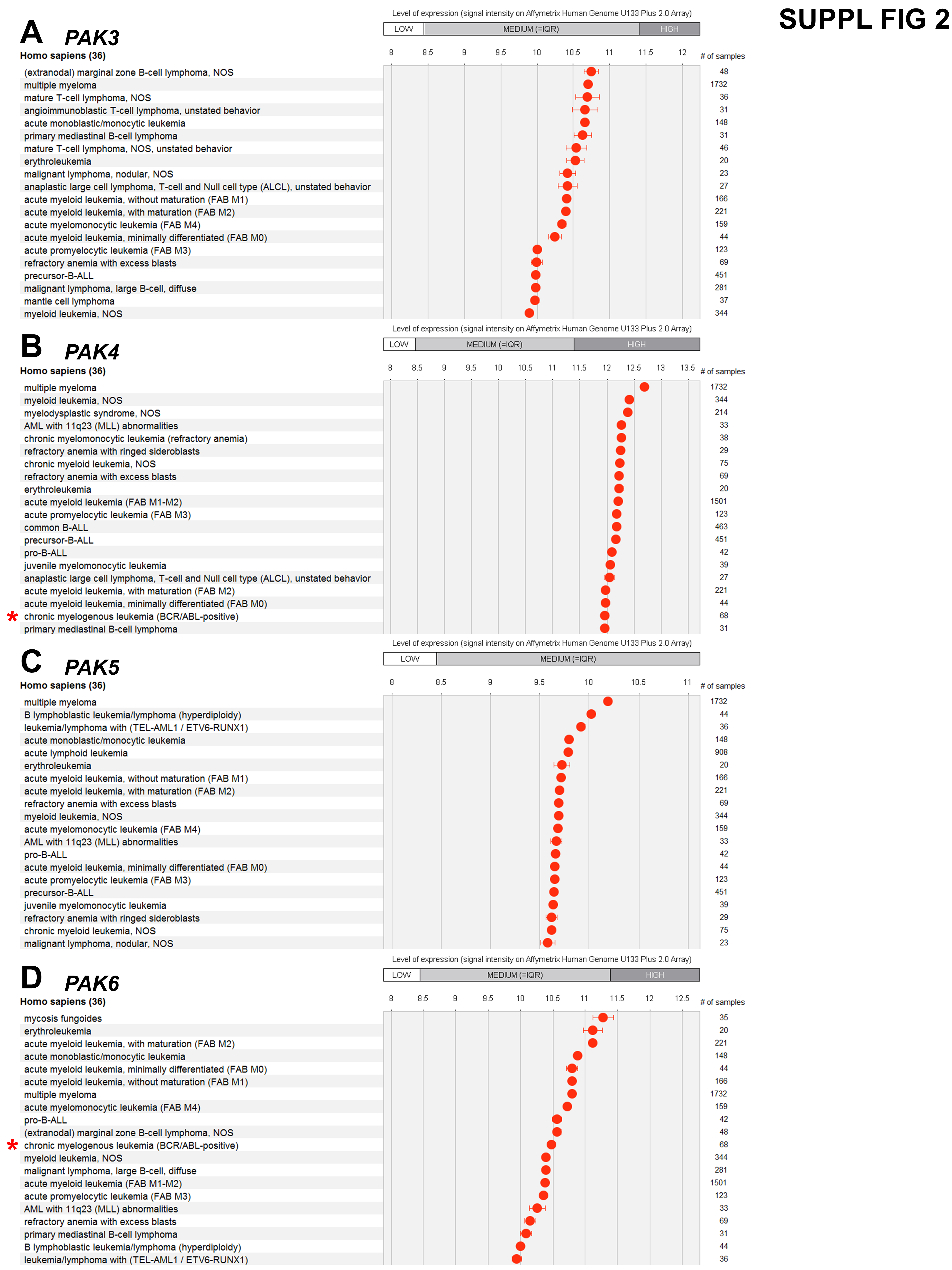

Supplement: Supplementary file 2 — Fig S2. Expression of PAK3, PAK4, PAK5 (7), and PAK6 in haematological diseases. [file BJH-179-229-s002.tif]

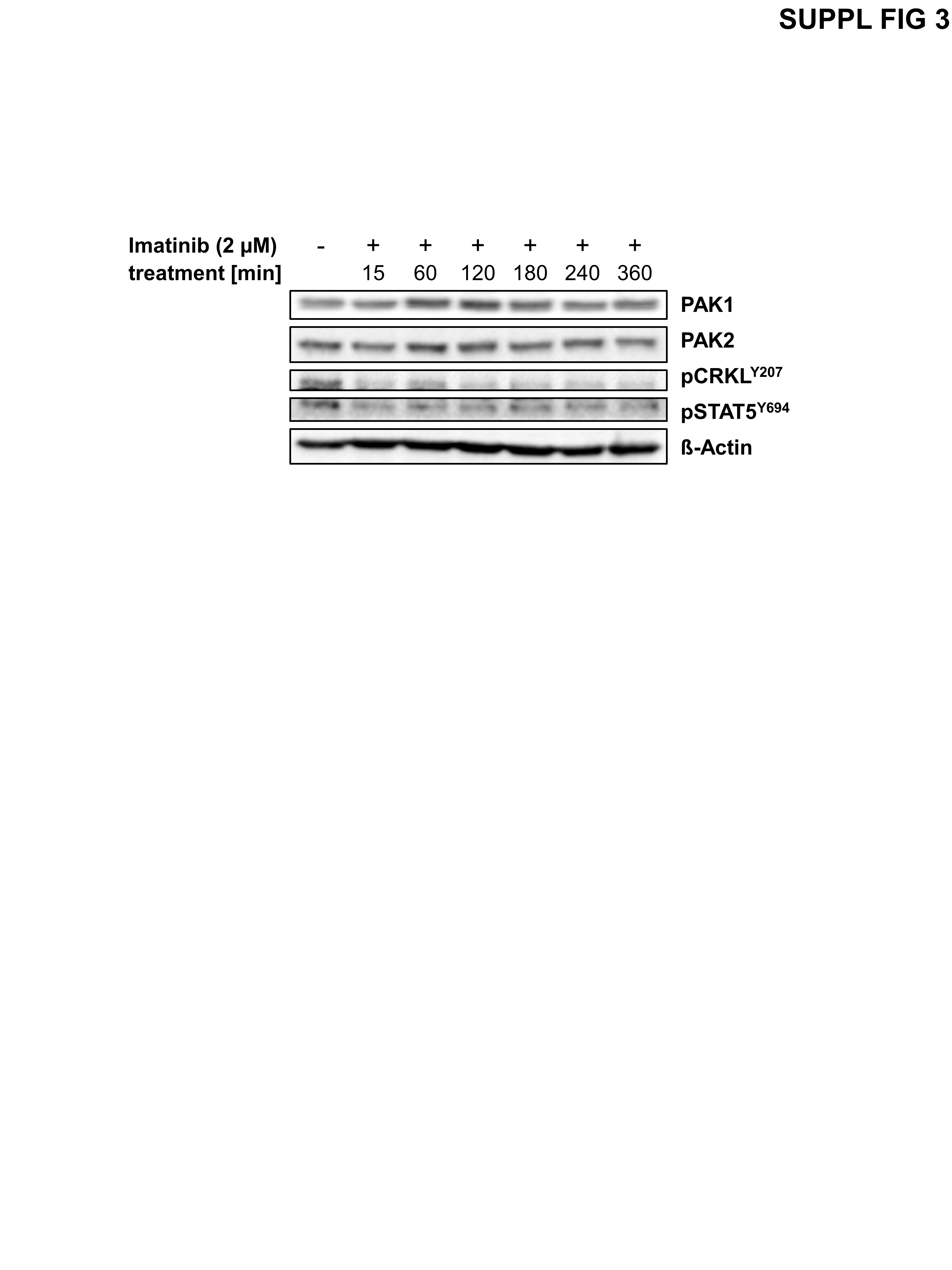

Supplement: Supplementary file 3 — Fig S3. Imatinib treatment of human BCR/ABL1 + KU812 cells. [file BJH-179-229-s003.tif]

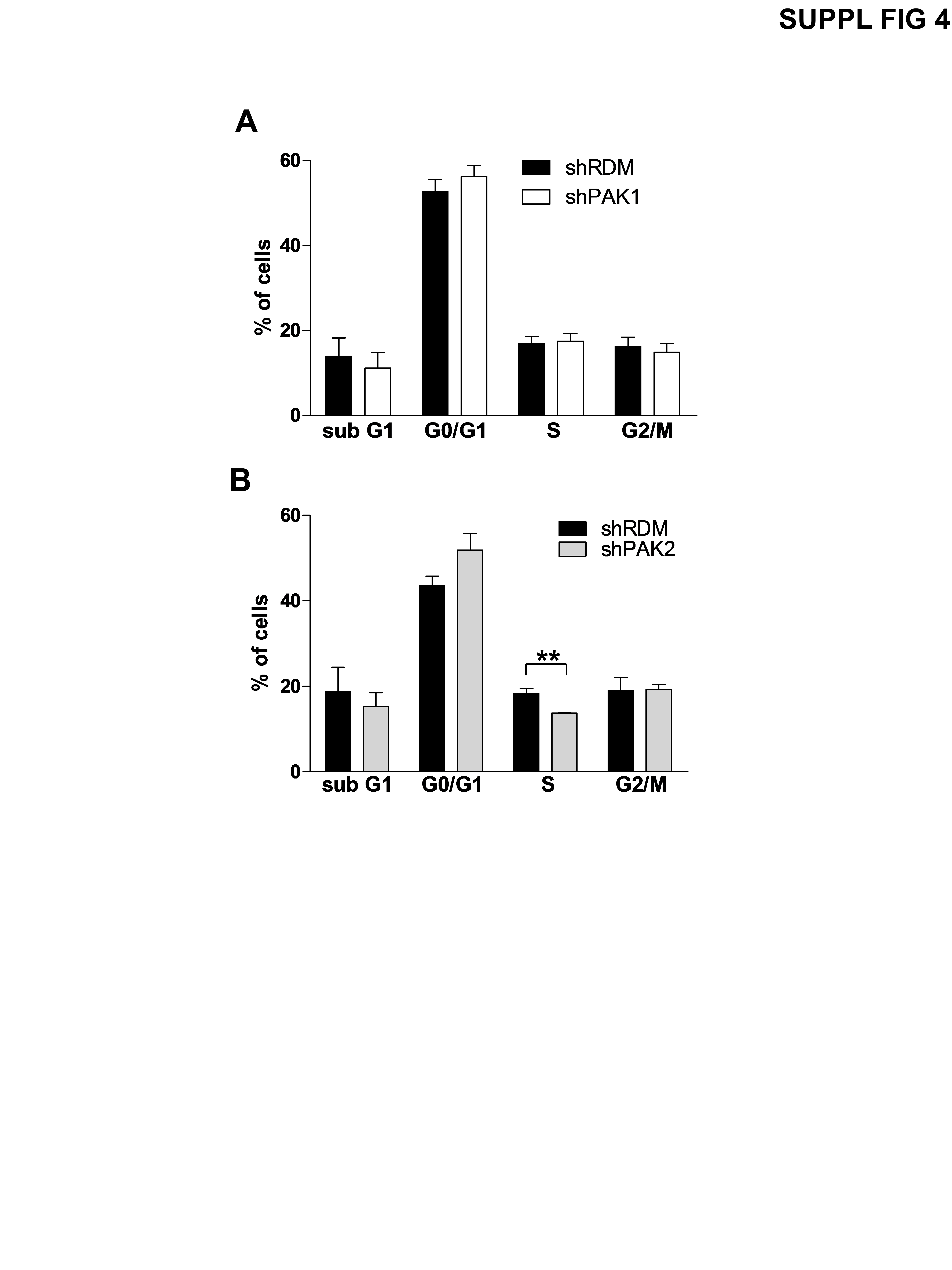

Supplement: Supplementary file 4 — Fig S4. Cell cycle analysis of human BCR/ABL1 + KU812 cells. [file BJH-179-229-s004.tif]

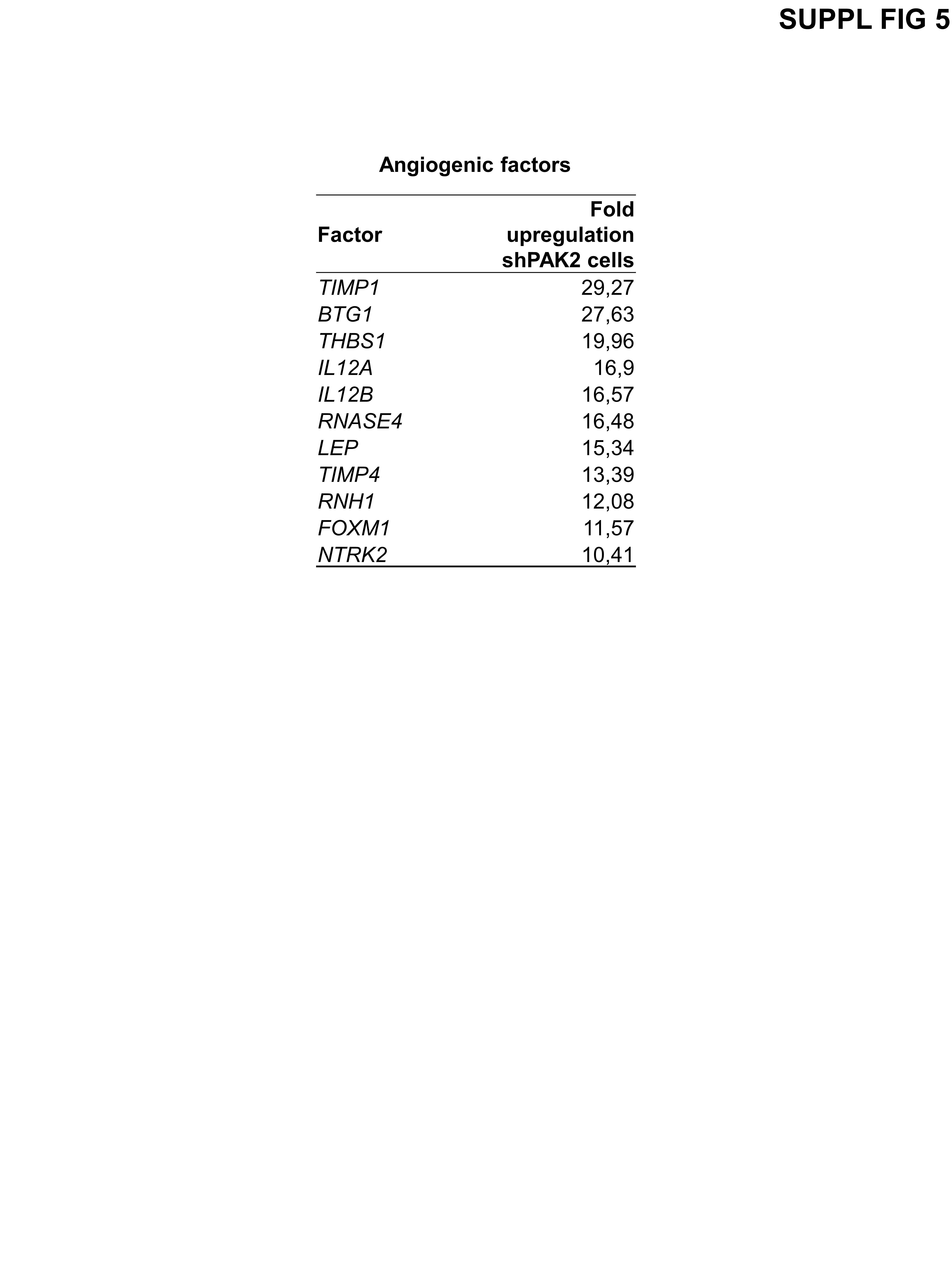

Supplement: Supplementary file 5 — Fig S5. Array for genes regulating angiogenesis. [file BJH-179-229-s005.tif]

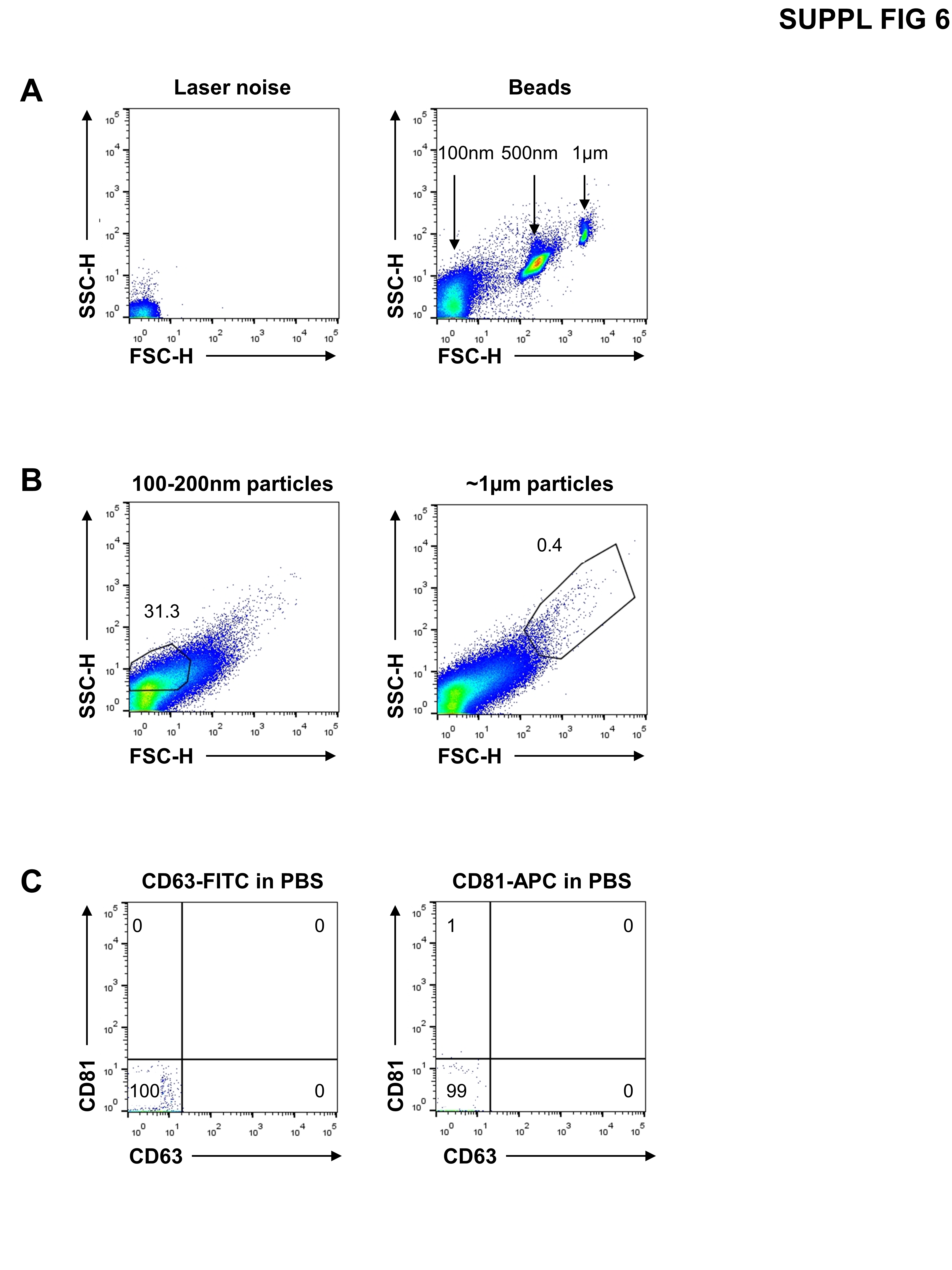

Supplement: Supplementary file 6 — Fig S6. Controls for setup of flow cytometry (exosome detection). [file BJH-179-229-s006.tif]
